# Perceiving being single as inherently negative: when participant ratings of another person’s life satisfaction rely solely on that person’s relationship status

**DOI:** 10.1186/s40359-025-03359-8

**Published:** 2025-09-26

**Authors:** Nikola Komlenac, Carmen Mair, Andreas Walther, Margarethe Hochleitner

**Affiliations:** 1 Institute for Diversity in Medicine, Medical University of Innsbruck, Innsbruck, Austria; 2 Psychotherapy Research, University of Graz, Graz, Austria; 3 Department of Clinical Psychology and Psychotherapy, University of Zurich, Zurich, Switzerland; 4 Innsbruck, Austria

**Keywords:** Singlism, Stereotypes, Public perception, German-speaking population

## Abstract

The belief that every person has a strong desire to be in a committed relationship can lead to stereotyping of single people. The present study examined whether people judge another person’s life satisfaction differently based solely on information about that person’s relationship status. We conducted a cross-sectional study among 1,241 participants (*M*_age_ = 40.3, *SD* = 13.0; 57.3% cisgender women, 40.8% cisgender men, and 1.9% gender minority individuals) in German-speaking countries. After reading one randomly assigned description of a character that included the character’s relationship status (single vs. with an uncommitted partner vs. with a committed partner), participants indicated how much they believed the character was satisfied with different aspects of life. Three informative hypotheses were compared using Bayesian ANCOVA. Results showed that participants rated characters with either an uncommitted or a committed partner to as having higher life satisfaction than single characters. Perceived life satisfaction was highest for characters with a committed partner. These judgements may reflect stereotypes and expectations that being single is inherently negative, which could ultimately contribute to discrimination or disadvantageous treatment of single people.

**Supplementary Information:**

The online version contains supplementary material available at 10.1186/s40359-025-03359-8.

## Introduction

In Great Britain 24% – 41% of the population reports being single [46], defined as “a subjective evaluation of an individual as a person who is not in a serious relationship” [1], p. 164). In Germany, a population-representative study found that single people make up to 36% of the population [7]. Single people vary in characteristics, interpersonal experiences [33], or motivations [60]. Single people’ life satisfaction is linked to those intra- and inter-individual factors [48, 76].

Many studies report lower life satisfaction among single people compared to those in committed relationships [10, 43]. However, a study conducted in Austria and Germany found that such differences in life satisfaction emerged only among individuals who were afraid of being single or of the prospect of becoming single [49]. Similarly, a large study in New Zealand and North America found that lower life satisfaction among single people was explained by their receiving less social support and experiencing more frequent negative treatment and discrimination compared to those in committed relationships [34].

### Singlism and committed relationship ideology

Singlism refers to negative treatment and discrimination toward single people, including stereotyping, rejection, or discrimination based on singlehood [18, 19]. In many contexts, being single past a certain age or not being in a committed relationship can be perceived negatively because it deviates from one aspect of heteronormative norms [74]. Heteronormativity prescribes expectations for women’s and men’s sexuality, assuming that people desire sexual involvement with someone of a different gender and to form committed relationships that ideally end in marriage [55].

Social structures that expect and privilege sexual and romantic attraction and relationships are also described as allonormativity [6]. Allonormativity and the committed relationship ideology prevail in many Western countries, including German-speaking regions of Europe, and reinforce the belief that almost everyone wants to be in a committed relationship [17]. Studies from North America and Europe show that allonormativity can lead to social exclusion, bullying, disaffirmation, and invalidation of asexual individuals (those who feel little or no sexual attraction) and aromantic individuals (those who do not experience romantic attraction; [5, 57, 81]). The committed relationship ideology can also affect allosexual and alloromantic individuals by promoting the belief that committed relationships are inherently good and central to happiness, while devaluing singlehood [16].

Internalized allonormativity and stereotypes can shape how perceivers judge others [45, 71]. Observers often view single people as having fewer favorable attributes or lower life satisfaction than people in a committed relationships [15, 47, 69]. For example, a vignette study in Germany presented short descriptions of characters [70] and found that participants perceived single characters as more miserable, lonelier, less warm and less caring than married characters [37].

Compared to singlehood, committed relationships are often associated with greater access to and more frequent interpersonal sexual activity [4, 63]. Uncommitted or casual relationships also tend to involve interpersonal sexual activity [54]. Higher frequency of interpersonal sexual activity is linked to greater life satisfaction [68]. However, a large U.S. study found that those who engaged in interpersonal sexual activity in the past year reported similar happiness levels as those who did not [47].

We expected participants to rate a character’s overall life satisfaction and satisfaction with sexual aspects of life as higher when the character was in a committed or uncommitted relationship, compared to being single, due to differences in access to and frequency of interpersonal sexual activity.

Committed relationships often come with greater social support [34] and lower levels of emotional loneliness, defined as the absence of an intimate relationship [10]. Singlehood, in contrast, is sometimes equated with loneliness, a subjective experience of lacking satisfying human relationships [64]. However, this assumption is not supported by research showing that many single people have broad social networks, including ties to relatives, neighbors, and friends [67], and often form meaningful and fulfilling emotional connections without feeling lonely [29].

We therefore examined whether a character’s relationship status influenced participants’ judgments of a third aspect of life satisfaction, namely perceived satisfaction with the character’s social life.

### Gendered expectations

Previous experiments on observers’ perceptions of single characters did not test whether single women are perceived differently from single men [37, 69]. Heteronormativity includes distinct gendered expectations and norms for women’s and men’s sexual behaviors, often referred to as sexual double standards [23, 44]. Men are often portrayed as more driven by sexual desire, and casual sexual activity is more accepted for them. In contrast, women are expected to be primarily interested in forming committed relationships [23, 44]. Consequently, singlism and negative perceptions of being single may affect women more strongly than men [32]. Women also report more negative feelings (e.g., worry, regret) about casual interpersonal sexual activity than men [78]. Furthermore, women who engage in such activity are often perceived as having low self-esteem, whereas men are not perceived this way [51]. Observers’ perceptions of single people engaging in interpersonal sexual activity with an uncommitted partner may therefore differ for women and men. We extend the literature by including vignettes describing characters who engage in interpersonal sexual activity with an uncommitted partner and by conducting separate analyses for female and male characters [37].

### The role of age in judging life satisfaction of single people

Heteronormativity includes expectations about when people should engage in certain sexual behaviors or enter committed relationships [55]. First interpersonal sexual experiences are often linked to growing up and becoming an adult [11, 12, 24]. Emerging adults who do not engage in interpersonal sexual activities or form committed relationships may therefore feel “off time” or “left behind” [21, 58, 59]. Based on this reasoning, we expected that information about a character’s relationship status would play a greater role in judging the life satisfaction of emerging adults (e.g., 25-year-old people) than of older adults, e.g., 50-year-old characters.

In contrast, the Developmental Life Tasks Model proposes that relationship status is more variable during emerging adulthood, but that with age, it becomes increasingly expected to be in a committed relationship [56]. According to this model, singlism would have a stronger impact on evaluations of 50-year-old people than on 25-year-old people. Previous research found that 40-year-old single people were judged to have more unfavorable attributes than 25-year-old single people [36]. In another study, single people were perceived as more miserable and lonely than married people, with this difference being larger for 40-year-old people than for people 25-year-old [37].

We examined whether harsher judgments of single characters would also emerge when comparing 50-year-old characters to 25-year-old characters. The Developmental Life Tasks Model predicts a greater difference in evaluations between single and partnered individuals at age 50 than at age 40 or 25 [56]. Therefore, we conducted separate analyses for 25-year-old and 50-year-old characters to assess occur in both age groups.

## Aim of the present study

We used vignettes, short depictions of hypothetical situations [27], consistent with a factorial survey approach [75], to examine whether participants’ judgments of a character’s life satisfaction and satisfaction with social and sexual aspects of life depended on the character’s relationship status. The short character descriptions were adapted from a previous study [37] and systematically varied by relationship status, age, and gender [31]. This design allowed us to test three hypotheses:

Research hypothesis 1: Ratings of life satisfaction and satisfaction with social and sexual aspects of life would be lowest for single characters, higher for characters in an uncommitted relationship, and highest for characters in a committed relationship.

Research hypothesis 2: The difference in perceived life satisfaction between characters with an uncommitted partner and those with a committed partner would be smaller for male than for female characters.

Research hypothesis 3: The difference in evaluations between single and partnered characters would be greater at age 50 than at age 25.

## Methods

### Participants and procedure

Recruitment took place between August and November 2022. We promoted the study through paid advertisements on Facebook and LinkedIn, targeting people over 18 years old living in Germany, Austria, or German-speaking regions of Italy [72] [66]. We also invited all students at an Austrian medical university via e-mail. The study was hosted on SoSci: der onlineFragebogen (SoSci Survey GmbH, Munich, Germany). Participants were told the study was about relationships but were not informed of its aims or hypotheses. Before beginning, participants gave consent for their anonymous data to be stored and used for research. No reimbursement was provided. The study followed the Declaration of Helsinki [80] and the APA standards [3]. On January 26, 2022, the Ethics Committee of the Medical University of Innsbruck exempted it from full review.

We included responses from 1,241 participants (57.3% cisgender women, 40.8% cisgender men, 1.9% gender minority individuals; age: *M* = 40.3 years, *SD* = 13.0, range: 18–80), after excluding 299 participants who failed two instructed response items (“Please select the response ‘Agree’”; [41, 77]). Table 1 shows the sample’s sociodemographic characteristics. Most participants were from Germany, identified as heterosexual, had an education at university entrance level or a university degree, and were in paid work. About half were single, and half were in a relationship. Participants with different sociodemographic characteristics were evenly distributed across vignette conditions (Table 1).

**Table 1:** Sociodemographic characteristics of the overall sample and by vignette condition

Variable		All *N* (%)	Single* N* (%)	Uncommitted *N* (%)	Committed	χ^2^	df
Whole sample		1241	385	430	426	2.5	4
Gender							
	Woman	711 (57.3)	212 (29.8)	246 (34.6)	253 (35.6)		
	Man	506 (40.8)	163 (32.2)	177 (35.0)	166 (32.8)		
	Gender minority	24 (1.9)	10 (41.7)	7 (29.2)	7 (29.2)		
Relationship						0.6	2
	Single	653 (52.6)	208 (31.9)	221 (33.8)	224 (34.3)		
	In relationship	588 (47.4)	177 (30.1)	209 (35.5)	202 (34.4)		
Nationality						0.7	4
	Austrian	292 (23.5)	95 (32.5)	99 (33.9)	98 (33.6)		
	German	785 (63.3)	237 (30.2)	276 (35.2)	272 (34.6)		
	Other	164 (13.2)	53 (32.3)	55 (33.5)	56 (34.1)		
Sexual Orientation						0.5	2
	Heterosexually identified	980 (79.0)	308 (31.4)	335 (34.2)	337 (34.4)		
	Sexual minority	261 (21.0)	77 (29.5)	95 (36.4)	89 (34.1)		
Education						3.5	4
	Primary school & vocational training	335 (27.5)	91 (27.2)	124 (37.0)	120 (35.8)		
	University entrance level	338 (27.8)	111 (32.8)	114 (33.7)	113 (33.4)		
	University degree	544 (44.7)	177 (32.5)	184 (33.8)	183 (33.6)		
Employment						6.4	4
	Working	906 (73.1)	273 (30.1)	331 (36.5)	302 (33.3)		
	Education	196 (15.8)	65 (33.2)	60 (30.6)	71 (36.2)		
	Not in paid work	137 (11.1)	47 (34.3)	37 (27.0)	53 (38.7)		

*df* degrees of freedom

### Measures

#### Vignettes

Participants read short descriptions of a character that included the character’s name, age, place of residence, relationship status, and interests to participants. These descriptions were adapted from Hertel et al. [37]. Participants were randomly assigned to one of 12 vignette conditions that could vary in the character’s name (2: name typically given to men or women), age (2: 25 or 50 years), and relationship status (3: single, in an uncommitted relationship with sexual activity, or in a committed relationship). All vignettes are provided in the Supplementary Material (S1 – S2).

#### Satisfaction with Life

We measured perceived life satisfaction using the Satisfaction with Life Scale [20]. The scale includes five statements (e.g., “In most ways my life is close to my ideal.”) rated on a seven-point Likert scale (1 = strongly disagree, 7 = strongly agree). Higher mean scores indicate higher life satisfaction. The original version showed an internal consistency of α = 0.87 [20]. We used the German version [35, 38], α = 0.89) and adapted the wording to refer to the vignette character rather than the participant. The modified version had an internal consistency of α = 0.87.

#### Self-satisfaction

The Self Satisfaction Scale (3S; [13]) assesses satisfaction with various life domains, including social relations, physical health, cognitive abilities, emotional and psychological well-being, physical appearance, sexual aspects (e.g., sexual attractiveness, flirting skills, sexual activity), and personality characteristics. We used only the subscales for social aspects (e.g., “I am satisfied with my social relations;” α = 0.90) and sexual aspects (e.g., “I am satisfied with myself in the field of sexuality;” α = 0.87). Two professional translators used the back-translation method [8] to translate the scales from English to German. We adapted the statements to refer to the vignette character rather than the participant. In this study, the social satisfaction subscale (five items) had an internal consistency of α = 0.87 and the sexual satisfaction subscale (three items) had an internal consistency of α = 0.87.

#### Sociodemographic information

We used self-constructed items to collect participants’ gender identity, age, sexual orientation, relationship status, highest level of education, employment status, and nationality. Participants selected from predefined options or provided free-text responses. All response options and sample characteristics are shown in Supplemental Table S3.

### Statistical analysis

We used SPSS for Windows, version 26.0 (IBM Corp., Armonk, NY, USA) to compute descriptive statistics. For life satisfaction, self-satisfaction with social aspects, and self-satisfaction with sexual aspects, we ran three separate Bayesian ANCOVAs, each with three informative hypotheses [39]:

1. Ratings do not differ by relationship status (μ_single_ = μ_uncommitted_ = μ_committed_).
2. Ratings are lowest for single characters, highest for characters in committed relationships, and intermediate for those in uncommitted relationships (μ_single_ < μ_uncommitted_ < μ_committed_).
3. Ratings are lower for single characters than for characters in any relationship, with equally high ratings for characters in uncommitted and committed relationships (μ_single_ < μ_uncommitted_ = μ_committed_).

We considered a hypothesis supported when the Bayes factor (BF0u) showed that the data were at least three times more likely under that hypothesis than under all alternatives (Hu; [39]). We also examined posterior model probabilities (PMPs) and interpreted a PMP ≥ 0.85 as substantial only when all competing hypotheses had low probabilities [39]. The Bayes Factor Matrix was used to compare relative support between hypotheses. We included participant age, gender, relationship status, nationality, sexual orientation, education, and employment, as well as the vignette character’s age and gender, as covariates in all Bayesian ANCOVAs. We also conducted ANCOVAs separately the character gender and age (Supplemental Tables S9, S15, S21, S27). We performed Bayesian ANCOVAs in JASP, version 0.16.3 (University of Amsterdam, Amsterdam, Netherlands). All subgroup comparisons exceeded the recommended minimum sample size of 121 for Bayesian hypothesis testing, with the smallest subgroup containing 189 participants [30],see Supplementary Material S8, S14, S20, S26).

## Results

### Descriptive statistics

On average, participants judged the character as being satisfied with their life (Table 2). Participants, who evaluated single characters, perceived them as neither satisfied nor dissatisfied with the social and sexual aspects of their life. Characters with an uncommitted partner were generally perceived as satisfied with both social and sexual aspects. Characters with a committed partner were perceived as satisfied with social aspects and as neither satisfied nor dissatisfied with sexual aspects (Table 2).

**Table 2:** Perception of the vignette characters’ satisfaction depending on relationship status – descriptive statistics

Variable	Single		Uncommitted			Committed		
	*M*	*SD*	*M*	*SD*	*d* (single vs. uncommitted)	*M*	*SD*	*d* (uncommitted vs. committed)
SWLS^1^	4.9	0.9	5.0	0.8	0.15	5.3	0.8	0.36
SSS social^2^	3.3	0.7	3.5	0.7	0.29	3.7	0.7	0.26
SSS sex^2^	2.7	0.8	3.8	0.8	1.36	3.4	0.8	0.48

*SWLS* Satisfaction with Life Scale, *SSS social* Self Satisfaction Scale – social, *SSS sex* Self Satisfaction Scale – sexual

^1^Possible range: 1 (not at all) – 7 (totally agree)

^2^Possible range: 1 (strongly disagree) – 5 (strongly agree)

Women gave higher satisfaction ratings for life, social, and sexual aspects, particularly when evaluating male characters and 50-year-old characters (Tables S7, S19, S31). In both the overall analysis the subgroup analyses by character gender, older characters received lower ratings for life, social, and sexual satisfaction (Tables S7, S13, S19). Participants with German or other nationalities gave lower ratings for social satisfaction than Austrian participants, especially when evaluating male characters and 25-year-old characters (Tables S7, S19, S25). Similarly, participants with German or other nationalities rated sexual satisfaction lower than Austrian participants when evaluating male characters (Table S19).

### Hypotheses testing

For life and social satisfaction, H2 (μ_single_ < μ_uncommitted_ < μ_committed_) received the strongest support (Table 3). Bayes factors from pairwise comparisons of the informative hypotheses (Supplemental Tables S4, S5) showed that support for H2 was more than 400 times greater than for H1 or H3.

**Table 3:** Probabilities that data fit the hypotheses

Hypothesis	SWLS		SSS social		SSS sex	
	Bf.u	PMP b	Bf.u	PMP b	Bf.u	PMP b
H1: μ_single_ = μ_uncommitted_ = μ_partnered_	< 0.01	<.01	< 0.01	< 0.01	< 0.01	< 0.01
H2: μ_single_ < μ_uncommitted_ < μ_partnered_	5.69	.85	5.82	.85	< 0.01	< 0.01
H3: μ_single_ < μ_uncommitted_ = μ_partnered_	< 0.01	<.01	0.01	< 0.01	< 0.01	< 0.01
Hu: all alternative hypotheses		.15		.15		> 0.99^a^

*SWLS* Satisfaction with Life Scale, *SSS social* Self Satisfaction Scale, *SSS sex* Self Satisfaction Scale – sexual, *Bf* Bayes factor, *PMP* posterior model probabilities

^a^An alternative hypothesis (H4: single < partnered < uncommitted) was tested and compared to Hu. H4 received 5.80 times (Bf.u) more support than did all alternative hypotheses (Hu) and the probability that H4 was the best hypothesis under investigation (while investigating H4 vs. Hu) was.85

None of the initial hypotheses (H1, H2, H3) were supported for ratings of the character’s sexual satisfaction (Table 3). Based on the descriptive results (Table 2), we formulated an alternative hypothesis, H4 (μ_single_ < μ_partnered_ < μ_uncommitted_), which predicted that ratings for the character’s sexual satisfaction would be lowest for single characters, highest for characters in uncommitted relationships, and intermediate for characters in committed relationships; this hypothesis was supported (Table 3).

#### Characters’ gender

H2 was supported for life satisfaction ratings of both female and male characters (Supplemental Tables S9, S15) and for social satisfaction ratings of female characters (Supplemental Table S9). Ratings of the male characters’ social satisfaction supported both H2 and H3 (μ_single_ < μ_uncommitted_ = μ_committed_; Supplemental Table S15).

For sexual satisfaction ratings of female and male characters, none of the initial hypotheses (H1, H2, H3) were supported. H4 received support in both cases (Supplemental Tables S9, S15).

#### Characters’ age

For 25-year-old characters, ratings of life and social satisfaction did not support H2 (Supplemental Table S21), as all alternative hypotheses (Hu) received considerable supported (16% – 26%). Using the descriptive results (Supplemental Table S20), we defined H5 (μ_single_ = μ_uncommitted_ < μ_partnered_), which predicted that ratings would be equally low for single characters and characters in uncommitted relationships, and higher for characters in committed relationships; this hypothesis was supported (Supplemental Table S21).

For 50-year-old characters, H2 was supported for life satisfaction ratings. Social satisfaction ratings supported both H2 and H3 (Supplemental Table S27).

For sexual satisfaction ratings of both 25-year-old and 50-year-old characters, none of the initial hypotheses (H1, H2, H3) was supported; H4 received support (Supplemental Tables S20, S21, S26, S27).

### Summary

#### Overall

Life and social satisfaction ratings were lowest for single characters, highest for those in committed relationships, and intermediate for those in uncommitted relationships. Sexual satisfaction ratings were lowest for single characters, highest for those in uncommitted relationships, and intermediate for those in committed relationships.

#### Female characters

Life and social satisfaction ratings followed the same pattern as overall results: lowest for singles, highest for committed, and intermediate for uncommitted. Sexual satisfaction ratings were lowest for single female characters, highest for uncommitted, and intermediate for committed.

#### Male characters

Life satisfaction ratings followed the overall pattern. Social satisfaction ratings were lower for single male characters than for those in any relationship. Sexual satisfaction ratings were lowest for single male characters, highest for uncommitted, and intermediate for committed.

#### 25-year-old characters

Life and social satisfaction ratings were equally low for single characters and uncommitted characters, and highest for committed characters. Sexual satisfaction ratings were lowest for singles, highest for uncommitted, and intermediate for committed.

#### 50-year-old characters

Life satisfaction ratings followed the overall pattern. Social satisfaction ratings were lower for single characters than for those in any relationship. Sexual satisfaction ratings were lowest for singles, highest for uncommitted, and intermediate for committed.

## Discussion

We included a vignette condition with characters in uncommitted relationships to explore whether perceptions of others’ satisfaction might rest on assumptions about their access to or engagement in interpersonal sexual activity. In addition to overall life satisfaction [36, 37], we examined specific domains — social and sexual satisfaction —because a committed partner is often viewed as a source of both [4, 34, 63].

Results supported Research Hypothesis 1: life and social satisfaction ratings were lowest for single characters, highest for those in committed relationships, and intermediate for those in uncommitted relationships. We found support for Research Hypothesis 2 because the difference in perceived social satisfaction between characters with an uncommitted partner and those with a committed partner was evident for female characters but not for male characters. For male characters, ratings for uncommitted and committed relationships were more similar, whereas for female characters, committed relationships were rated more favorably, highlighting the stronger perceived importance of commitment for women. Findings did not support Research Hypothesis 3. Life and social satisfaction ratings were equally low for 25-year-old single characters and those in uncommitted relationships, and highest for those in committed relationships. This pattern reflects heteronormative assumptions about the importance of committed relationships in emerging adulthood [55] rather than predictions based on the Developmental Life Tasks Model [56]. Consistently, single people who have internalized such assumptions might experience greater contentment with being single as they age [61].

Across all subgroups, single characters consistently received the lowest life satisfaction ratings in every domain. This pattern likely reflects participants’ internalized allonormativity and stereotypes [45, 71]. Our findings align with previous research showing that people perceive single individuals as lonelier [37], and less satisfied with life [10, 43]. However, such perceptions do not necessarily match single individuals’ self-reports and overlook the heterogeneity within this group in terms of personal characteristics, interpersonal experiences [33], and other factors that shape life satisfaction [48, 53, 73, 76]. Negative stereotypes and preconceptions about a social group can lead to discrimination or unequal treatment of its members [28, 62]. Single individuals report lower levels of social support and more frequent negative treatment and discrimination than people in committed relationships [34]. Asexual and aromantic individuals may also face discrimination when they do not desire or engage in romantic or sexual relationships [5, 42]. Prejudice and stereotypes about single individuals often remain more publicly acceptable than those about other groups, such as sexual minorities [28]. Being subjected to prejudice or discrimination is linked to lower self-esteem [9] and reduced life satisfaction [34].

Our findings suggest that perceptions of others’ satisfaction may be based on assumptions about their access to or engagement in interpersonal sexual activity, as well as on the belief that single people who do not engage in such activity cannot be as satisfied as those who do. These assumptions can be inaccurate and may not reflect the lived experiences of single individuals [47]. In fact, many people who are voluntarily single and have no desire to form committed relationships report being satisfied with the sexual aspects of their lives [2, 48, 52, 73].

A distinct pattern emerged for sexual satisfaction ratings: they were highest for characters in uncommitted relationships, followed by those in committed relationships, and lowest for single characters. This pattern was similar for both women and men. However, past research shows that women often face devaluation and stigmatization for engaging in casual interpersonal sexual activity [32]. Many women internalize this stigma and experience guilt or shame, which has been linked to poorer mental health outcomes [78]. Sexual double standards can therefore discourage or penalize women for engaging in sexual activities that, overall, are associated with higher sexual satisfaction ratings [78].

### Implications

Based on our findings, we emphasize the importance of deconstructing the culturally reinforced link between “growing up” and forming a committed relationship. Prior research suggests that many emerging adults who believe that achieving the milestones of “becoming an adult” depends on engaging in interpersonal sexual activity or entering a committed relationship may feel pressured to do so out of fear of being “off time” or “left behind” [21, 58, 59]. This pressure can increase their risk for negative sexual health outcomes [50]. We agree with the suggestion that, during formal education, adolescents and emerging adults should be provided with a “social and psychological ‘toolbox’” that helps them learn how to be a satisfied single person [48], p. 735). This includes learning about sexuality and different ways to have a fulfilling sexual life while single, as well as understanding the diverse lifestyles of single people. Furthermore, healthcare workers, therapists, and coaches should avoid applying the committed relationship ideology. They should recognize that single people are a diverse demographic group with varying inter- and intra-individual characteristics, and that these characteristics — rather than relationship status — are linked to life satisfaction [48, 76].

### Limitations

No consensus exists on a cut-off value for posterior model probabilities (PMPs). We followed the recommendation of considering a PMP ≥ 0.85 as substantial [39]. This threshold may appear very different from the alpha error of 0.05 commonly used in statistical testing [26]. However, in this study we tested three hypotheses, and PMPs tend to decrease as the number of defined hypotheses increases [39]. A key advantage of Bayesian ANCOVA was the ability to test the plausibility and relative support for H1 — the hypothesis that no difference existed between the vignette conditions (often referred to as the null hypothesis). All PMPs for H1 were far below 0.01, indicating strong evidence against it. These results support the conclusion that single characters and characters with an uncommitted or a committed partner were perceived differently.

Participants received relatively little information about the vignette characters. This limited context may have made it difficult to judge the characters’ satisfaction in various life domains. It remains unclear which specific preconceptions or prior knowledge participants relied on for their judgments. Some participants may expect people to achieve certain “milestones” to be satisfied with life, with forming a committed relationship being one such milestone [21]. However, we did not assess the beliefs underlying participants’ ratings. Future research could use qualitative methods, such as the think-aloud method [22], to explore the thoughts and emotions the vignettes trigger, especially those contributing to unfavorable perceptions of singlehood.

As with many questionnaire studies, we relied on self-reports. Participants may have provided inaccurate responses or aimed to respond in socially desirable ways [14]. Although vignettes are believed to reduce socially desirable responding [79], future studies should ask participants whether they assumed other participants were given different vignette conditions, and whether they formed specific hypotheses about the expected study results. Such expectations may have influenced their responses.

## Conclusion

Participants’ ratings may reflect internalized relationship ideologies and allonormative expectations [45, 71], as the vignettes differed only in the character’s relationship status [6]. Across all subgroups, single characters consistently received the lowest ratings for life satisfaction in every domain. Such preconceptions — often not aligned with single individuals’ self-reports or experiences [33] — can contribute to negative stereotypes about single people [81] and, ultimately, to discrimination or unequal treatment [5, 28, 62].

Future research should explore how negative stereotypes or preconceptions lead to differential or unfavorable treatment of single people. For example, studies could examine how physicians respond upon learning of a patient’s singlehood during sexual history taking [65]. It would also be important to investigate whether communications across various channels (e.g., media, everyday conversation) contain negative messaging about singlehood. Understanding the links between such messages, adherence to the committed relationship ideology, and fear of being or becoming single may reveal a critical factor influencing sexual health.

## Supplementary Information

Supplementary Material 1

## Data Availability

The datasets used and/or analyzed in the present study are available from the corresponding author on reasonable request.
