# Supplementary material for: Perceiving being single as inherently negative: when participant ratings of another person’s life satisfaction rely solely on that person’s relationship status
Source: BMC Psychol. 2025 Sep 26;13:1034. doi: 10.1186/s40359-025-03359-8 (PMC12466009; doi:10.1186/s40359-025-03359-8)
Supplement: Supplementary file 1 — Supplementary Material 1 [file 40359_2025_3359_MOESM1_ESM.docx]

**S1**

*Vignettes – German*

Magdalena/Martin ist 25/50 Jahre alt. Er/Sie lebt in München und arbeitet als Labortechniker*in. Er/Sie ist single/in einer festen Beziehung/in einer unverbindlichen Beziehungen (mit sexueller Aktivität). In der Freizeit interessiert sich Martin/Magdalena für Musik und Sport.

**Man, young, single**

Martin ist 25 Jahre alt. Er lebt in München und arbeitet als Labortechniker*in. Er ist single. In der Freizeit interessiert sich Martin für Musik und Sport.

**Man, young, uncommitted**

Martin ist 25 Jahre alt. Er lebt in München und arbeitet als Labortechniker*in. Er ist in einer unverbindlichen Beziehungen (mit sexueller Aktivität). In der Freizeit interessiert sich Martin für Musik und Sport.

**Man, young, committed**

Martin ist 25 Jahre alt. Er lebt in München und arbeitet als Labortechniker*in. Er ist in einer festen Beziehung. In der Freizeit interessiert sich Martin für Musik und Sport.

**Man, old, single**

Martin ist 50 Jahre alt. Er lebt in München und arbeitet als Labortechniker*in. Er ist single. In der Freizeit interessiert sich Martin für Musik und Sport.

**Man, old, uncommitted**

Martin ist 50 Jahre alt. Er lebt in München und arbeitet als Labortechniker*in. Er ist in einer unverbindlichen Beziehungen (mit sexueller Aktivität). In der Freizeit interessiert sich Martin für Musik und Sport.

**Man, old, committed**

Martin ist 50 Jahre alt. Er lebt in München und arbeitet als Labortechniker*in. Er ist in einer festen Beziehung. In der Freizeit interessiert sich Martin für Musik und Sport.

**Woman, young, single**

Magdalena ist 25 Jahre alt. Sie lebt in München und arbeitet als Labortechniker*in. Sie ist single. In der Freizeit interessiert sich Magdalena für Musik und Sport.

**Woman, young, uncommitted**

Magdalena ist 25 Jahre alt. Sie lebt in München und arbeitet als Labortechniker*in. Sie ist in einer unverbindlichen Beziehungen (mit sexueller Aktivität). In der Freizeit interessiert sich Magdalena für Musik und Sport.

**Woman, young, committed**

Magdalena ist 25 Jahre alt. Sie lebt in München und arbeitet als Labortechniker*in. Er ist in einer festen Beziehung. In der Freizeit interessiert sich Magdalena für Musik und Sport.

**Woman, old, single**

Magdalena ist 50 Jahre alt. Sie lebt in München und arbeitet als Labortechniker*in. Sie ist single. In der Freizeit interessiert sich Magdalena für Musik und Sport.

**Woman, old, uncommitted**

Magdalena ist 50 Jahre alt. Sie lebt in München und arbeitet als Labortechniker*in. Sie ist in einer unverbindlichen Beziehungen (mit sexueller Aktivität). In der Freizeit interessiert sich Magdalena für Musik und Sport.

**Woman, old, committed**

Magdalena ist 50 Jahre alt. Sie lebt in München und arbeitet als Labortechniker*in. Sie ist in einer festen Beziehung. In der Freizeit interessiert sich Magdalena für Musik und Sport.

**S2**

*Vignettes – English*

Magdalena/Martin is 25/50 years old. He/She lives in Munich and works as a laboratory technician. He/She is single/in a committed relationship/in an uncommitted relationship (with sexual activity). Martin/Magdalena is interested in music and sports.

**Man, young, single**

Martin is 25 years old. He lives in Munich and works as a laboratory technician. He is single. Martin is interested in music and sports.

**Man, young, uncommitted**

Martin is 25 years old. He lives in Munich and works as a laboratory technician. He is in an uncommitted relationship (with sexual activity). Martin is interested in music and sports.

**Man, young, committed**

Martin is 25 years old. He lives in Munich and works as a laboratory technician. He is in a committed relationship. Martin is interested in music and sports.

**Man, old, single**

Martin is 50 years old. He lives in Munich and works as a laboratory technician. He is single. Martin is interested in music and sports.

**Man, old, uncommitted**

Martin is 50 years old. He lives in Munich and works as a laboratory technician. He is in an uncommitted relationship (with sexual activity). Martin is interested in music and sports.

**Man, old, committed**

Martin is 50 years old. He lives in Munich and works as a laboratory technician. He is in a committed relationship. Martin is interested in music and sports.

**Woman, young, single**

Magdalena is 25 years old. She lives in Munich and works as a laboratory technician. She is single. Magdalena is interested in music and sports.

**Woman, young, uncommitted**

Magdalena is 25 years old. She lives in Munich and works as a laboratory technician. She is in an uncommitted relationship (with sexual activity). Magdalena is interested in music and sports.

**Woman, young, committed**

Magdalena is 25 years old. She lives in Munich and works as a laboratory technician. She is in a committed relationship. Magdalena is interested in music and sports.

**Woman, old, single**

Magdalena is 50 years old. She lives in Munich and works as a laboratory technician. She is single. Magdalena is interested in music and sports.

**Woman, old, uncommitted**

Martin is 50 years old. She lives in Munich and works as a laboratory technician. She is in an uncommitted relationship (with sexual activity). Magdalena is interested in music and sports.

**Woman, old, committed**

Magdalena is 50 years old. She lives in Munich and works as a laboratory technician. She is in a committed relationship. Magdalena likes music and sports.

**S3**

*Sociodemographic Description of the Sample*

| Variable | | All *N* (%) | Category for analysis |
| --- | --- | --- | --- |
| Gender |  |  |  |
|  | Woman | 711 (57.3) | Woman |
|  | Man | 506 (40.8) | Man |
|  | Trans*man | 4 (0.3) | Gender minority |
|  | Trans*woman | 5 (0.4) |  |
|  | Nonbinary | 10 (0.8) |  |
|  | Divers | 4 (0.3) |  |
|  | Other | 1 (0.1) |  |
| Relationship |  |  |  |
|  | Single | 648 (52.2) | Single |
|  | In relationship with sexual activity | 407 (32.8) | In relationship |
|  | In relationship without sexual activity | 32 (2.6) |  |
|  | Open relationship | 105 (8.5) |  |
|  | Relationship with more than one partner | 20 (1.6) |  |
|  | Other with open text response | 20 (1.6) | 5 🡪 single 15 🡪 in relationship |
| Nationality |  |  |  |
|  | Austrian | 292 (23.5) | Austrian |
|  | German | 785 (63.3) | German |
|  | Swiss | 28 (2.3) | Other |
|  | Italian | 121 (9.8) |  |
|  | Other | 15 (1.2) |  |
| Sexual Orientation |  |  |  |
|  | Heterosexually identified | 979 (79.8) | Heterosexually identified |
|  | Gay-identified/lesbian-identified | 50 (4.1) | Sexual minority |
|  | Bisexual-identified | 118 (9.6) |  |
|  | Asexual-identified | 22 (1.8) |  |
|  | Pansexual-identified | 47 (3.8) |  |
|  | Other | 11 (0.9) | 1 🡪 heterosexually identified 10 🡪 sexual minority |
| Education |  |  |  |
|  | Primary school | 108 (8.9) | Primary school & vocational training |
|  | Vocational training | 227 (18.7) |  |
|  | University entrance level | 338 (27.8) | University entrance level |
|  | University degree | 544 (44.7) | University degree |
| Employment |  |  |  |
|  | Working | 897 (72.3) | Working |
|  | Education | 194 (15.6) | Education |
|  | Unemployed | 33 (2.7) | Not in paid work |
|  | Retirement | 81 (6.5) |  |
|  | Parental leave | 6 (0.5) |  |
|  | Other | 30 (2.4) | 9 🡪 working 2 🡪 education 15 🡪 not in paid work |

*Note*. df = degrees of freedom

**S4**

*Bayes Factors between Pairs of Informative Hypotheses for the Variable Satisfaction with Life (SWLS)*

|  | H1 | H2 | H3 |
| --- | --- | --- | --- |
| H1: μ_single_ = μ_uncommitted_ = μ_partnered_ | 1.00 | 0.00 | 0.00 |
| H2: μ_single_ < μ_uncommitted_ < μ_partnered_ | > 10,000.00^a^ | 1.00 | > 10,000.00 |
| H3: μ_single_ < μ_uncommitted_ = μ_partnered_ | > 10,000.00 | 0.00 | 1.00 |

^a^ Support for H2 is over 10,000.00 times larger than for H1

**S5**

*Bayes Factors between Pairs of Informative Hypotheses for the Variable Self-satisfaction with Social Aspects (SSS social)*

|  | H1 | H2 | H3 |
| --- | --- | --- | --- |
| H1: μ_single_ = μ_uncommitted_ = μ_partnered_ | 1.00 | 0.00 | 0.00 |
| H2: μ_single_ < μ_uncommitted_ < μ_partnered_ | > 10,000.00 | 1.00 | 464.03 |
| H3: μ_single_ < μ_uncommitted_ = μ_partnered_ | > 10,000.00 | 0.00 | 1.00 |

**S6**

*Bayes Factors between Pairs of Informative Hypotheses for the Self-satisfaction with Sexual Aspects (SSS sex)*

|  | H1 | H2 | H3 |
| --- | --- | --- | --- |
| H1: μ_single_ = μ_uncommitted_ = μ_partnered_ | 1.00 | 0.00 | 0.00 |
| H2: μ_single_ < μ_uncommitted_ < μ_partnered_ | > 10,000.00 | 1.00 | 0.01 |
| H3: μ_single_ < μ_uncommitted_ = μ_partnered_ | > 10,000.00 | 146.99 | 1.00 |

**S7**

*All Factor Coefficients*

|  |  | Coefficient | SE | LL | UL |
| --- | --- | --- | --- | --- | --- |
| SWLS |  |  |  |  |  |
|  | Age | -0.01 | 0.00 | -0.01 | 0.00 |
|  | Gender | 0.15* | 0.05 | 0.05 | 0.24 |
|  | Relationship | -0.07 | 0.05 | -0.16 | 0.03 |
|  | Nationality | -0.06 | 0.04 | -0.14 | 0.02 |
|  | Sexual Orientation | 0.02 | 0.06 | -0.10 | 0.14 |
|  | Education | 0.03 | 0.03 | -0.03 | 0.09 |
|  | Employment | 0.03 | 0.04 | -0.04 | 0.10 |
|  | Character’s age | -0.18* | 0.05 | -0.28 | -0.09 |
|  | Character’s gender | -0.01 | 0.05 | -0.11 | 0.08 |
| SSS social |  |  |  |  |  |
|  | Age | 0.00 | 0.00 | 0.00 | 0.00 |
|  | Gender | 0.12* | 0.04 | 0.05 | 0.20 |
|  | Relationship | -0.07 | 0.04 | -0.15 | 0.01 |
|  | Nationality | -0.09* | 0.03 | -0.16 | -0.03 |
|  | Sexual Orientation | 0.06 | 0.05 | -0.04 | 0.15 |
|  | Education | 0.01 | 0.02 | -0.04 | 0.06 |
|  | Employment | -0.05 | 0.03 | -0.11 | 0.01 |
|  | Character’s age | -0.14* | 0.04 | -0.22 | -0.06 |
|  | Character’s gender | 0.07 | 0.04 | -0.01 | 0.14 |
| SSS sex |  |  |  |  |  |
|  | Age | 0.00 | 0.00 | 0.00 | 0.00 |
|  | Gender | 0.13* | 0.04 | 0.04 | 0.22 |
|  | Relationship | -0.09 | 0.05 | -0.18 | 0.00 |
|  | Nationality | -0.08 | 0.04 | -0.15 | 0.00 |
|  | Sexual Orientation | 0.01 | 0.06 | -0.10 | 0.12 |
|  | Education | 0.01 | 0.03 | -0.05 | 0.06 |
|  | Employment | 0.02 | 0.03 | -0.05 | 0.08 |
|  | Character’s age | -0.19* | 0.05 | -0.28 | -0.10 |
|  | Character’s gender | 0.05 | 0.05 | -0.04 | 0.14 |

*Note.* SWLS = Satisfaction with Life Scale; SSS social = Self Satisfaction Scale; SSS sex = Self Satisfaction Scale – sexual

**S8**

*Perception of the Main Characters’ Satisfaction Depending on Relationship Status – Descriptive Statistics – Magdalena*

| Variable | Single (*n* = 196) | | Uncommitted (*n* = 215) | | | Committed (*n* = 210) | | |
| --- | --- | --- | --- | --- | --- | --- | --- | --- |
|  | *M* | *SD* | *M* | *SD* | *d* (single vs. uncommitted) | *M* | *SD* | *d* (uncommitted vs. committed) |
| SWLS^1^ | 4.9 | 0.9 | 5.1 | 0.8 | 0.18 | 5.4 | 0.8 | 0.35 |
| SSS social^2^ | 3.4 | 0.7 | 3.5 | 0.6 | 0.20 | 3.7 | 0.7 | 0.28 |
| SSS sex^2^ | 2.7 | 0.8 | 3.8 | 0.8 | 1.35 | 3.4 | 0.8 | 0.48 |

*Note.* ^1^Possible range: 1 (not at all) – 7 (totally agree); ^2^Possible range: 1 (strongly disagree) – 5 (strongly agree);
SWLS = Satisfaction with Life Scale; SSS social = Self Satisfaction Scale – social; SSS sex = Self Satisfaction Scale – sexual

**S9**

*Probabilities that Data Fit the Hypotheses – Magdalena*

| Hypothesis | SWLS | | SSS social | | SSS sex | |
| --- | --- | --- | --- | --- | --- | --- |
|  | Bf.u | PMP b | Bf.u | PMP b | Bf.u | PMP b |
| H1: μ_single_ = μ_uncommitted_ = μ_partnered_ | 0.00 | < .01 | < 0.01 | .01 | < 0.01 | < 0.01 |
| H2: μ_single_ < μ_uncommitted_ < μ_partnered_ | 5.62 | .83 | 5.53 | .80 | < 0.01 | < 0.01 |
| H3: μ_single_ < μ_uncommitted_ = μ_partnered_ | 0.16 | .02 | 0.42 | .06 | < 0.01 | < 0.01 |
| Hu: all alternative hypotheses |  | .15 |  | .14 |  | > 0.99^a^ |

*Note.* ^a^An alternative hypothesis (H4: single < partnered < uncommitted) was tested and compared to Hu. H4 received 5.81 times (Bf.u) more support than did all alternative hypotheses (Hu) and the probability that H4 was the best hypothesis under investigation (while investigating H4 vs. Hu) was .85.
SWLS = Satisfaction with Life Scale; SSS social = Self Satisfaction Scale; SSS sex = Self Satisfaction Scale – sexual; Bf = Bayes factor; PMP = posterior model probabilities

**S10**

*Bayes Factors between Pairs of Informative Hypotheses for the Variable Satisfaction with Life (SWLS) – Magdalena*

|  | H1 | H2 | H3 |
| --- | --- | --- | --- |
| H1: μ_single_ = μ_uncommitted_ = μ_partnered_ | 1.00 | 0.00 | 0.00 |
| H2: μ_single_ < μ_uncommitted_ < μ_partnered_ | 9594.11 | 1.00 | 34.33 |
| H3: μ_single_ < μ_uncommitted_ = μ_partnered_ | 279.48 | 0.03 | 1.00 |

^a^ Support for H2 is over 10,000.00 times larger than for H1

**S11**

*Bayes Factors between Pairs of Informative Hypotheses for the Variable Self-satisfaction with Social Aspects (SSS social) – Magdalena*

|  | H1 | H2 | H3 |
| --- | --- | --- | --- |
| H1: μ_single_ = μ_uncommitted_ = μ_partnered_ | 1.00 | 0.00 | 0.01 |
| H2: μ_single_ < μ_uncommitted_ < μ_partnered_ | 915.15 | 1.00 | 13.23 |
| H3: μ_single_ < μ_uncommitted_ = μ_partnered_ | 69.16 | 0.08 | 1.00 |

**S12**

*Bayes Factors between Pairs of Informative Hypotheses for the Self-satisfaction with Sexual Aspects (SSS sex) – Magdalena*

|  | H1 | H2 | H3 |
| --- | --- | --- | --- |
| H1: μ_single_ = μ_uncommitted_ = μ_partnered_ | 1.00 | 0.00 | 0.00 |
| H2: μ_single_ < μ_uncommitted_ < μ_partnered_ | > 10,000.00^a^ | 1.00 | 0.01 |
| H3: μ_single_ < μ_uncommitted_ = μ_partnered_ | > 10,000.00 | 78.46 | 1.00 |

**S13**

*All factor coefficients – Magdalena*

|  |  | Coefficient | SE | LL | UL |
| --- | --- | --- | --- | --- | --- |
| SWLS |  |  |  |  |  |
|  | Age | -0.01* | 0.00 | -0.02 | -0.01 |
|  | Gender | 0.11 | 0.07 | -0.03 | 0.24 |
|  | Relationship | -0.05 | 0.07 | -0.19 | 0.08 |
|  | Nationality | -0.01 | 0.06 | -0.12 | 0.11 |
|  | Sexual Orientation | 0.02 | 0.08 | -0.15 | 0.18 |
|  | Education | 0.05 | 0.04 | -0.03 | 0.14 |
|  | Employment | 0.04 | 0.06 | -0.07 | 0.15 |
|  | Character’s age | -0.20* | 0.07 | -0.34 | -0.07 |
| SSS social |  |  |  |  |  |
|  | Age | 0.00 | 0.00 | -0.01 | 0.00 |
|  | Gender | 0.07 | 0.05 | -0.03 | 0.18 |
|  | Relationship | -0.04 | 0.05 | -0.15 | 0.06 |
|  | Nationality | -0.09 | 0.05 | -0.18 | 0.00 |
|  | Sexual Orientation | 0.07 | 0.07 | -0.06 | 0.20 |
|  | Education | 0.02 | 0.03 | -0.04 | 0.09 |
|  | Employment | -0.07 | 0.04 | -0.16 | 0.01 |
|  | Character’s age | -0.13* | 0.05 | -0.24 | -0.03 |
| SSS sex |  |  |  |  |  |
|  | Age | 0.00 | 0.00 | -0.01 | 0.00 |
|  | Gender | 0.09 | 0.06 | -0.03 | 0.21 |
|  | Relationship | -0.14* | 0.06 | -0.26 | -0.01 |
|  | Nationality | -0.02 | 0.05 | -0.12 | 0.09 |
|  | Sexual Orientation | 0.01 | 0.08 | -0.14 | 0.16 |
|  | Education | 0.04 | 0.04 | -0.04 | 0.11 |
|  | Employment | -0.02 | 0.05 | -0.12 | 0.08 |
|  | Character’s age | -0.18* | 0.06 | -0.31 | -0.06 |

**S14**

*Perception of the Main Characters’ Satisfaction Depending on Relationship Status – Descriptive Statistics – Martin*

| Variable | Single (*n* = 189) | | Uncommitted (*n* = 215) | | | Committed (*n* = 216) | | |
| --- | --- | --- | --- | --- | --- | --- | --- | --- |
|  | *M* | *SD* | *M* | *SD* | *d* (single vs. uncommitted) | *M* | *SD* | *d* (uncommitted vs. committed) |
| SWLS^1^ | 4.9 | 0.9 | 5.0 | 0.8 | 0.10 | 5.3 | 0.9 | 0.36 |
| SSS social^2^ | 3.2 | 0.7 | 3.5 | 0.7 | 0.38 | 3.7 | 0.8 | 0.24 |
| SSS sex^2^ | 2.6 | 0.9 | 3.7 | 0.8 | 1.38 | 3.4 | 0.8 | 0.48 |

*Note.* ^1^Possible range: 1 (not at all) – 7 (totally agree); ^2^Possible range: 1 (strongly disagree) – 5 (strongly agree);
SWLS = Satisfaction with Life Scale; SSS social = Self Satisfaction Scale – social; SSS sex = Self Satisfaction Scale – sexual

**S15**

*Probabilities that Data Fit the Hypotheses – Martin*

| Hypothesis | SWLS | | SSS social | | SSS sex | |
| --- | --- | --- | --- | --- | --- | --- |
|  | Bf.u | PMP b | Bf.u | PMP b | Bf.u | PMP b |
| H1: μ_single_ = μ_uncommitted_ = μ_partnered_ | < 0.01 | < .01 | < 0.01 | < .01 | < 0.01 | < 0.01 |
| H2: μ_single_ < μ_uncommitted_ < μ_partnered_ | 5.01 | .83 | 5.79 | .71 | < 0.01 | < 0.01 |
| H3: μ_single_ < μ_uncommitted_ = μ_partnered_ | 0.02 | < .01 | 1.36 | .17 | < 0.01 | < 0.01 |
| Hu: all alternative hypotheses |  | .17 |  | .12 |  | > 0.99^a^ |

*Note.* ^a^An alternative hypothesis (H4: μ_single_ < μ_partnered_ < μ_uncommitted_) was tested and compared to Hu. H4 received 5.81 times (Bf.u) more support than did all alternative hypotheses (Hu) and the probability that H4 was the best hypothesis under investigation (while investigating H4 vs. Hu) was .85.
SWLS = Satisfaction with Life Scale; SSS social = Self Satisfaction Scale; SSS sex = Self Satisfaction Scale – sexual; Bf = Bayes factor; PMP = posterior model probabilities

**S16**

*Bayes Factors between Pairs of Informative Hypotheses for the Variable Satisfaction with Life (SWLS) – Martin*

|  | H1 | H2 | H3 |
| --- | --- | --- | --- |
| H1: μ_single_ = μ_uncommitted_ = μ_partnered_ | 1.00 | 0.00 | 0.02 |
| H2: μ_single_ < μ_uncommitted_ < μ_partnered_ | > 10,000.00 | 1.00 | 300.66 |
| H3: μ_single_ < μ_uncommitted_ = μ_partnered_ | 41.70 | 0.00 | 1.00 |

**S17**

*Bayes Factors between Pairs of Informative Hypotheses for the Variable Self-satisfaction with Social Aspects (SSS social) – Martin*

|  | H1 | H2 | H3 |
| --- | --- | --- | --- |
| H1: μ_single_ = μ_uncommitted_ = μ_partnered_ | 1.00 | 0.00 | 0.00 |
| H2: μ_single_ < μ_uncommitted_ < μ_partnered_ | > 10,000.00 | 1.00 | 4.27 |
| H3: μ_single_ < μ_uncommitted_ = μ_partnered_ | > 10,000.00 | 0.23 | 1.00 |

**S18**

*Bayes Factors between Pairs of Informative Hypotheses for the Self-satisfaction with Sexual Aspects (SSS sex) – Martin*

|  | H1 | H2 | H3 |
| --- | --- | --- | --- |
| H1: μ_single_ = μ_uncommitted_ = μ_partnered_ | 1.00 | 0.00 | 0.00 |
| H2: μ_single_ < μ_uncommitted_ < μ_partnered_ | > 10,000.00 | 1.00 | 0.01 |
| H3: μ_single_ < μ_uncommitted_ = μ_partnered_ | > 10,000.00 | 76.58 | 1.00 |

**S19**

*All factor coefficients – Martin*

|  |  | Coefficient | SE | LL | UL |
| --- | --- | --- | --- | --- | --- |
| SWLS |  |  |  |  |  |
|  | Age | 0.00 | 0.00 | -0.01 | 0.00 |
|  | Gender | 0.18* | 0.07 | 0.05 | 0.32 |
|  | Relationship | -0.08 | 0.07 | -0.22 | 0.06 |
|  | Nationality | -0.11 | 0.06 | -0.22 | 0.00 |
|  | Sexual Orientation | 0.01 | 0.09 | -0.16 | 0.18 |
|  | Education | 0.01 | 0.04 | -0.08 | 0.09 |
|  | Employment | 0.02 | 0.05 | -0.08 | 0.12 |
|  | Character’s age | -0.18* | 0.07 | -0.32 | -0.04 |
| SSS social |  |  |  |  |  |
|  | Age | 0.00 | 0.00 | 0.00 | 0.01 |
|  | Gender | 0.19* | 0.06 | 0.08 | 0.30 |
|  | Relationship | -0.11 | 0.06 | -0.22 | 0.01 |
|  | Nationality | -0.10* | 0.05 | -0.19 | -0.01 |
|  | Sexual Orientation | 0.05 | 0.07 | -0.10 | 0.19 |
|  | Education | 0.00 | 0.04 | -0.07 | 0.07 |
|  | Employment | -0.03 | 0.04 | -0.11 | 0.05 |
|  | Character’s age | -0.17* | 0.06 | -0.28 | -0.06 |
| SSS sex |  |  |  |  |  |
|  | Age | 0.00 | 0.00 | 0.00 | 0.01 |
|  | Gender | 0.17* | 0.06 | 0.04 | 0.29 |
|  | Relationship | -0.05 | 0.07 | -0.18 | 0.08 |
|  | Nationality | -0.13* | 0.05 | -0.24 | -0.03 |
|  | Sexual Orientation | 0.01 | 0.08 | -0.15 | 0.17 |
|  | Education | -0.02 | 0.04 | -0.10 | 0.06 |
|  | Employment | 0.05 | 0.05 | -0.04 | 0.14 |
|  | Character’s age | -0.22* | 0.07 | -0.35 | -0.09 |

**S20**

*Perception of the Main Characters’ Satisfaction Depending on Relationship Status – Descriptive Statistics – 25-year Old Characters*

| Variable | Single (*n* = 200) | | Uncommitted (*n* = 205) | | | Committed (*n* = 213) | | |
| --- | --- | --- | --- | --- | --- | --- | --- | --- |
|  | *M* | *SD* | *M* | *SD* | *d* (single vs. uncommitted) | *M* | *SD* | *d* (uncommitted vs. committed) |
| SWLS^1^ | 5.1 | 0.9 | 5.1 | 0.8 | 0.02 | 5.4 | 0.9 | 0.35 |
| SSS social^2^ | 3.4 | 0.7 | 3.5 | 0.6 | 0.16 | 3.7 | 0.7 | 0.29 |
| SSS sex^2^ | 2.8 | 0.9 | 3.7 | 0.8 | 1.17 | 3.5 | 0.8 | 0.28 |

*Note.* ^1^Possible range: 1 (not at all) – 7 (totally agree); ^2^Possible range: 1 (strongly disagree) – 5 (strongly agree);
SWLS = Satisfaction with Life Scale; SSS social = Self Satisfaction Scale – social; SSS sex = Self Satisfaction Scale – sexual

**S21**

*Probabilities that Data Fit the Hypotheses – 25-year Old Characters*

| Hypothesis | SWLS | | SSS social | | SSS sex | |
| --- | --- | --- | --- | --- | --- | --- |
|  | Bf.u | PMP b | Bf.u | PMP b | Bf.u | PMP b |
| H1: μ_single_ = μ_uncommitted_ = μ_partnered_ | 0.01 | 0.00 | 0.01 | < 0.01 | 0.00 | < 0.01 |
| H2: μ_single_ < μ_uncommitted_ < μ_partnered_ | 2.81 | 0.73^a^ | 5.20 | 0.82^b^ | 0.03 | 0.01 |
| H3: μ_single_ < μ_uncommitted_ = μ_partnered_ | 0.01 | 0.00 | 0.13 | 0.02 | 1.48 | 0.59^c^ |
| Hu: all alternative hypotheses |  | 0.26 |  | 0.16 |  | 0.40 |

*Note.* ^a^An alternative hypothesis (H5: μ_single_ = μ_uncommitted_ < μ_partnered_) was tested and compared to Hu. H5 reiceived 34.58 times (Bf.u) more support than did all alternative hypotheses (Hu) and the probability that H5 was the best hypothesis under investigation (while investigating H5 vs. Hu) was .97.
^b^An alternative hypothesis (H5: μ_single_ = μ_uncommitted_ < μ_partnered_) was tested and compared to Hu. H5 received 15.66 times (Bf.u) more support than did all alternative hypotheses (Hu) and the probability that H5 was the best hypothesis under investigation (while investigating H5 vs. Hu) was .94.
^c^An alternative hypothesis (H4: μ_single_ < μ_partnered_ < μ_uncommitted_) was tested and compared to Hu. H4 received 5.75 times (Bf.u) more support than did all alternative hypotheses (Hu) and the probability that H4 was the best hypothesis under investigation (while investigating H4 vs. Hu) was .85.
SWLS = Satisfaction with Life Scale; SSS social = Self Satisfaction Scale; SSS sex = Self Satisfaction Scale – sexual; Bf = Bayes factor; PMP = posterior model probabilities

**S22**

*Bayes Factors between Pairs of Informative Hypotheses for the Variable Satisfaction with Life (SWLS) – 25-year Old Characters*

|  | H1 | H2 | H3 |
| --- | --- | --- | --- |
| H1: μ_single_ = μ_uncommitted_ = μ_partnered_ | 1.00 | 0.00 | 1.00 |
| H2: μ_single_ < μ_uncommitted_ < μ_partnered_ | 238.80 | 1.00 | 238.36 |
| H3: μ_single_ < μ_uncommitted_ = μ_partnered_ | 1.00 | 0.00 | 1.00 |

**S23**

*Bayes Factors between Pairs of Informative Hypotheses for the Variable Self-satisfaction with Social Aspects (SSS social) – 25-year Old Characters*

|  | H1 | H2 | H3 |
| --- | --- | --- | --- |
| H1: μ_single_ = μ_uncommitted_ = μ_partnered_ | 1.00 | 0.00 | 0.05 |
| H2: μ_single_ < μ_uncommitted_ < μ_partnered_ | 789.64 | 1.00 | 39.52 |
| H3: μ_single_ < μ_uncommitted_ = μ_partnered_ | 19.98 | 0.03 | 1.00 |

**S24**

*Bayes Factors between Pairs of Informative Hypotheses for the Self-satisfaction with Sexual Aspects (SSS sex) – 25-year Old Characters*

|  | H1 | H2 | H3 |
| --- | --- | --- | --- |
| H1: μ_single_ = μ_uncommitted_ = μ_partnered_ | 1.00 | 0.00 | 0.00 |
| H2: μ_single_ < μ_uncommitted_ < μ_partnered_ | > 10,000.00 | 1.00 | 0.02 |
| H3: μ_single_ < μ_uncommitted_ = μ_partnered_ | > 10,000.00 | 43.83 | 1.00 |

**S25**

*All factor coefficients– 25-year Old Characters*

|  |  | Coefficient | SE | LL | UL |
| --- | --- | --- | --- | --- | --- |
| SWLS |  |  |  |  |  |
|  | Age | -0.01* | 0.00 | -0.02 | -0.01 |
|  | Gender | 0.12 | 0.07 | -0.01 | 0.26 |
|  | Relationship | -0.12 | 0.07 | -0.25 | 0.01 |
|  | Nationality | -0.06 | 0.06 | -0.17 | 0.05 |
|  | Sexual Orientation | -0.03 | 0.09 | -0.19 | 0.14 |
|  | Education | -0.02 | 0.04 | -0.10 | 0.06 |
|  | Employment | 0.02 | 0.05 | -0.08 | 0.12 |
|  | Character’s gender | -0.01 | 0.07 | -0.14 | 0.12 |
| SSS social |  |  |  |  |  |
|  | Age | 0.00 | 0.00 | -0.01 | 0.00 |
|  | Gender | 0.02 | 0.06 | -0.09 | 0.13 |
|  | Relationship | -0.07 | 0.06 | -0.18 | 0.04 |
|  | Nationality | -0.13* | 0.05 | -0.22 | -0.03 |
|  | Sexual Orientation | 0.01 | 0.07 | -0.13 | 0.15 |
|  | Education | -0.03 | 0.03 | -0.10 | 0.03 |
|  | Employment | -0.06 | 0.04 | -0.14 | 0.03 |
|  | Character’s gender | 0.05 | 0.06 | -0.06 | 0.16 |
| SSS sex |  |  |  |  |  |
|  | Age | 0.00 | 0.00 | -0.01 | 0.01 |
|  | Gender | 0.01 | 0.06 | -0.12 | 0.13 |
|  | Relationship | -0.10 | 0.07 | -0.22 | 0.03 |
|  | Nationality | -0.09 | 0.05 | -0.20 | 0.02 |
|  | Sexual Orientation | 0.03 | 0.08 | -0.13 | 0.19 |
|  | Education | -0.01 | 0.04 | -0.09 | 0.07 |
|  | Employment | 0.00 | 0.05 | -0.09 | 0.10 |
|  | Character’s gender | 0.04 | 0.06 | -0.09 | 0.16 |

**S26**

*Perception of the Main Characters’ Satisfaction Depending on Relationship Status – Descriptive Statistics – 50-year Old Characters*

| Variable | Single (*n* = 200) | | Uncommitted (*n* = 205) | | | Committed (*n* = 213) | | |
| --- | --- | --- | --- | --- | --- | --- | --- | --- |
|  | *M* | *SD* | *M* | *SD* | *d* (single vs. uncommitted) | *M* | *SD* | *d* (uncommitted vs. committed) |
| SWLS^1^ | 4.8 | 0.9 | 5.0 | 0.9 | 0.24 | 5.3 | 0.8 | 0.36 |
| SSS social^2^ | 3.2 | 0.6 | 3.5 | 0.7 | 0.40 | 3.6 | 0.7 | 0.23 |
| SSS sex^2^ | 2.5 | 0.8 | 3.8 | 0.8 | 1.55 | 3.3 | 0.8 | 0.68 |

*Note.* ^1^Possible range: 1 (not at all) – 7 (totally agree); ^2^Possible range: 1 (strongly disagree) – 5 (strongly agree);
SWLS = Satisfaction with Life Scale; SSS social = Self Satisfaction Scale – social; SSS sex = Self Satisfaction Scale – sexual

**S27**

*Probabilities that Data Fit the Hypotheses – 50-year Old Characters*

| Hypothesis | SWLS | | SSS social | | SSS sex | |
| --- | --- | --- | --- | --- | --- | --- |
|  | Bf.u | PMP b | Bf.u | PMP b | Bf.u | PMP b |
| H1: μ_single_ = μ_uncommitted_ = μ_partnered_ | 0.00 | < 0.01 | 0.00 | < 0.01 | < 0.01 | < 0.01 |
| H2: μ_single_ < μ_uncommitted_ < μ_partnered_ | 5.77 | 0.85 | 5.79 | 0.72 | < 0.01 | < 0.01 |
| H3: μ_single_ < μ_uncommitted_ = μ_partnered_ | 0.06 | 0.01 | 1.31 | 0.16 | < 0.01 | < 0.01 |
| Hu: all alternative hypotheses |  | 0.15 |  | 0.12 |  | > 0.99^a^ |

*Note.* ^a^An alternative hypothesis (H4: μ_single_ < μ_partnered_ < μ_uncommitted_) was tested and compared to Hu. H4 received 5.77 times (Bf.u) more support than did all alternative hypotheses (Hu) and the probability that H4 was the best hypothesis under investigation (while investigating H4 vs. Hu) was .85.
SWLS = Satisfaction with Life Scale; SSS social = Self Satisfaction Scale; SSS sex = Self Satisfaction Scale – sexual; Bf = Bayes factor; PMP = posterior model probabilities

**S28**

*Bayes Factors between Pairs of Informative Hypotheses for the Variable Satisfaction with Life (SWLS) – 50-year Old Characters*

|  | H1 | H2 | H3 |
| --- | --- | --- | --- |
| H1: μ_single_ = μ_uncommitted_ = μ_partnered_ | 1.00 | 0.00 | 0.00 |
| H2: μ_single_ < μ_uncommitted_ < μ_partnered_ | > 10,000.00 | 1.00 | 99.05 |
| H3: μ_single_ < μ_uncommitted_ = μ_partnered_ | > 10,000.00 | 0.01 | 1.00 |

**S29**

*Bayes Factors between Pairs of Informative Hypotheses for the Variable Self-satisfaction with Social Aspects (SSS social) – 50-year Old Characters*

|  | H1 | H2 | H3 |
| --- | --- | --- | --- |
| H1: μ_single_ = μ_uncommitted_ = μ_partnered_ | 1.00 | 0.00 | 0.00 |
| H2: μ_single_ < μ_uncommitted_ < μ_partnered_ | > 10,000.00 | 1.00 | 4.41 |
| H3: μ_single_ < μ_uncommitted_ = μ_partnered_ | > 10,000.00 | 0.23 | 1.00 |

**S30**

*Bayes Factors between Pairs of Informative Hypotheses for the Self-satisfaction with Sexual Aspects (SSS sex) – 50-year Old Characters*

|  | H1 | H2 | H3 |
| --- | --- | --- | --- |
| H1: μ_single_ = μ_uncommitted_ = μ_partnered_ | 1.00 | 0.00 | 0.00 |
| H2: μ_single_ < μ_uncommitted_ < μ_partnered_ | > 10,000.00 | 1.00 | 0.01 |
| H3: μ_single_ < μ_uncommitted_ = μ_partnered_ | > 10,000.00 | 105.15 | 1.00 |

**S31**

*All factor coefficients– 50-year Old Characters*

|  |  | Coefficient | SE | LL | UL |
| --- | --- | --- | --- | --- | --- |
| SWLS |  |  |  |  |  |
|  | Age | -0.01 | 0.00 | -0.01 | 0.00 |
|  | Gender | 0.16* | 0.07 | 0.03 | 0.29 |
|  | Relationship | -0.03 | 0.07 | -0.17 | 0.11 |
|  | Nationality | -0.06 | 0.06 | -0.18 | 0.06 |
|  | Sexual Orientation | 0.04 | 0.09 | -0.13 | 0.21 |
|  | Education | 0.07 | 0.04 | -0.01 | 0.16 |
|  | Employment | 0.04 | 0.05 | -0.06 | 0.15 |
|  | Character’s gender | -0.02 | 0.07 | -0.16 | 0.12 |
| SSS social |  |  |  |  |  |
|  | Age | 0.00 | 0.00 | -0.01 | 0.00 |
|  | Gender | 0.22* | 0.05 | 0.12 | 0.32 |
|  | Relationship | -0.08 | 0.06 | -0.19 | 0.03 |
|  | Nationality | -0.06 | 0.05 | -0.15 | 0.03 |
|  | Sexual Orientation | 0.08 | 0.07 | -0.05 | 0.22 |
|  | Education | 0.05 | 0.03 | -0.02 | 0.12 |
|  | Employment | -0.05 | 0.04 | -0.13 | 0.04 |
|  | Character’s gender | 0.08 | 0.06 | -0.03 | 0.19 |
| SSS sex |  |  |  |  |  |
|  | Age | 0.00 | 0.00 | -0.01 | 0.00 |
|  | Gender | 0.23* | 0.06 | 0.11 | 0.35 |
|  | Relationship | -0.10 | 0.06 | -0.23 | 0.02 |
|  | Nationality | -0.06 | 0.05 | -0.17 | 0.04 |
|  | Sexual Orientation | -0.03 | 0.08 | -0.19 | 0.13 |
|  | Education | 0.03 | 0.04 | -0.05 | 0.10 |
|  | Employment | 0.03 | 0.05 | -0.07 | 0.12 |
|  | Character’s gender | 0.06 | 0.06 | -0.06 | 0.19 |
